# The Bile Salt Export Pump: Molecular Structure, Study Models and Small-Molecule Drugs for the Treatment of Inherited BSEP Deficiencies

**DOI:** 10.3390/ijms22020784

**Published:** 2021-01-14

**Authors:** Muhammad Imran Sohail, Yaprak Dönmez-Cakil, Dániel Szöllősi, Thomas Stockner, Peter Chiba

**Affiliations:** 1 Department of Zoology, Government College University, Lahore 54000, Pakistan; imransohail@gcu.edu.pk; 2 Department of Histology and Embryology, Faculty of Medicine, Maltepe University, Maltepe, 34857 Istanbul, Turkey; yaprak.cakil@maltepe.edu.tr; 3 Institute of Pharmacology, Center for Physiology and Pharmacology, Medical University of Vienna, Waehringerstrasse, 13A, 1090 Vienna, Austria; daniel.szoelloesi@meduniwien.ac.at; 4 Institute of Medical Chemistry, Center for Pathobiochemistry and Genetics, Medical University of Vienna, Waehringerstrasse, 10, 1090 Vienna, Austria

**Keywords:** BSEP, ABCB11, bile salts, intrahepatic cholestasis, chaperones, PFIC2, BRIC

## Abstract

The bile salt export pump (BSEP/ABCB11) is responsible for the transport of bile salts from hepatocytes into bile canaliculi. Malfunction of this transporter results in progressive familial intrahepatic cholestasis type 2 (PFIC2), benign recurrent intrahepatic cholestasis type 2 (BRIC2) and intrahepatic cholestasis of pregnancy (ICP). Over the past few years, several small molecular weight compounds have been identified, which hold the potential to treat these genetic diseases (chaperones and potentiators). As the treatment response is mutation-specific, genetic analysis of the patients and their families is required. Furthermore, some of the mutations are refractory to therapy, with the only remaining treatment option being liver transplantation. In this review, we will focus on the molecular structure of ABCB11, reported mutations involved in cholestasis and current treatment options for inherited BSEP deficiencies.

## 1. Introduction

**Figure 1:**
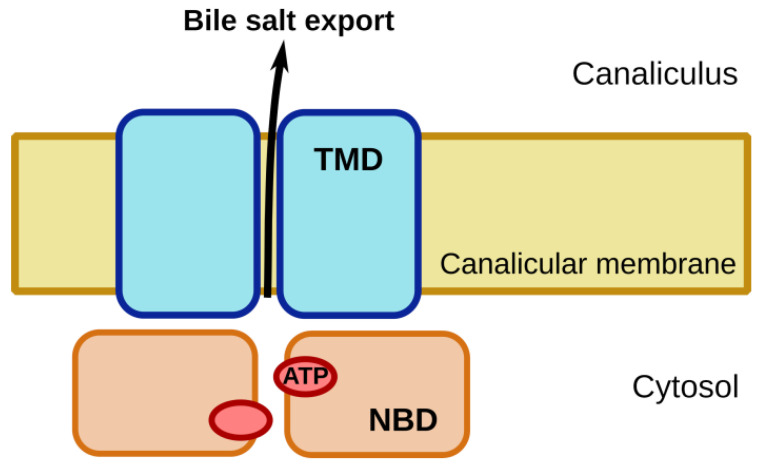
Schematic of the four-domain architecture of an ATP-binding cassette (ABC) transporter, as illustrated for the bile salt export pump BSEP. Hydrolysis of ATP by the nucleotide binding domains (NBD) energizes transport of bile salts from the hepatocyte cytoplasm to the bile canaliculus. Transmembrane domains (TMD) form the path for bile salt transport across the canalicular membrane.

The ATP-binding cassette (ABC) proteins constitute one of the largest families of membrane proteins. They are universally present in all kingdoms of life. In humans, 48 functional genes encode for ABC proteins, which on the basis of structural and sequence similarity are categorized into seven subfamilies, designated as ABCA through G [1]. Most of these proteins transport substrates across cellular membranes. A functional ABC transporter comprises at least four domains: two transmembrane domains (TMDs) and two nucleotide-binding domains (NBDs), as shown in Figure 1. In the ABCB subfamily, each of the TMDs consists of six membrane-spanning helices. Five of these extend into the cytoplasm to form an expansive intracellular domain. The two TMDs are responsible for substrate binding and translocation. The NBDs form two composite nucleotide-binding sites (NBSs) at their interface, which bind and hydrolyze ATP, and thereby provide the energy for substrate transport. These NBSs are formed by the Walker A and Walker B motifs, as well as the A-, Q- and H-loops of one NBD and the signature motif and D-loop of the other NBD [2,3].

The ABCB subfamily is one of the most diverse groups of ABC proteins, as it includes dimeric half transporters, monomers of which are each composed of one TMD and one NBD, but also full-length transporters, in which all four domains are fused into a single polypeptide chain. The former group comprises the homodimeric transporters ABCB6, ABCB7, ABCB8, ABCB9 and ABCB10, and the heterodimeric transporter ABCB2/ABCB3. The four full-length transporters of the ABCB subfamily are ABCB1, ABCB4, ABCB5 and ABCB11. The heterodimeric ABCB2/ABCB3 and full-length ABCB11 differ from the other members as they contain only one canonical NBS rather than two [4].

## 2. BSEP/ABCB11: Physiological Role

The bile salt export pump (BSEP) is expressed in hepatocytes. While high levels of BSEP mRNA were detected in testes and lower levels were reported in other extrahepatic tissues, including trachea, prostate, lungs, thymus, kidney and colon [5,6], plasma membrane expression of functional protein was found in liver cells only [5,6,7,8]. Adjacent hepatocytes form tight junctions to enclose functional structures called bile canaliculi, to which BSEP is targeted. Bile salts undergo an enterohepatic circulation, which depends on active transport systems in the liver and intestine. In the course of this process, newly synthesized and recycled bile salts are secreted from hepatocytes into bile canaliculi by BSEP and via bile ducts reach the duodenum. In the ileum, these bile salts are reabsorbed by the apical sodium-dependent bile salt transporter in intestinal epithelial cells (ASBT/SLC10A2). From the intestine, bile salts return to the liver via the superior mesenteric and portal veins, which carry the blood that feeds liver sinusoids. Uptake into hepatocytes is mediated by the sodium taurocholate co-transporting polypeptide (NTCP/SLC10A1) and organic anion transporters (OATPs). Bile salt transport by BSEP constitutes the rate-limiting step in bile formation and provides the major driving force for enterohepatic circulation [9].

The bile salt pool is recycled from the intestine to the liver six to eight times a day [10], resulting in daily bile salt excretion of about 20–40 g [11]. Impairment of BSEP results in the failure to maintain physiological bile flow, resulting in a clinical condition called intrahepatic cholestasis. BSEP has narrow specificity for its substrate bile salts, but the drugs pravastatin, vinblastine and fexofenadine are reported to be non-physiological substrates [12,13,14].

## 3. Transcriptional Regulation

BSEP expression is highly regulated by transcriptional mechanisms, and a wide inter-individual variability has been described at the mRNA and protein levels [15].

**Figure 2:**
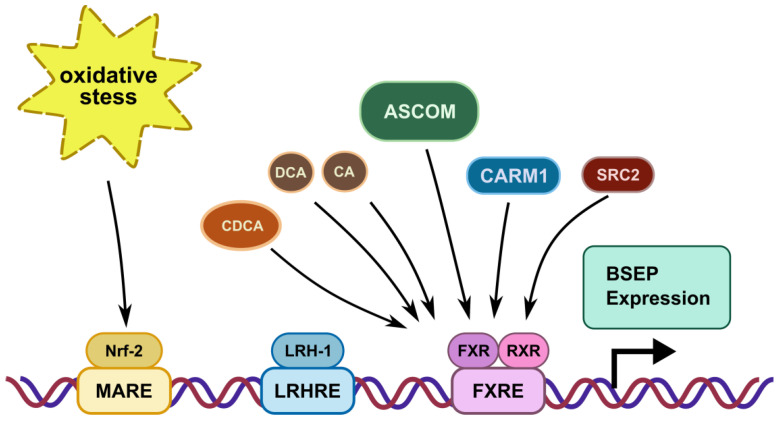
Transcriptional regulation of BSEP via recognition sequences in the promoter region of the BSEP gene: MARE: Maf recognition element; LRHRE: liver receptor homolog-1 responsive element; FXRE: farnesoid X receptor responsive element. The farnesoid receptor FXR binds bile salts after heterodimerization with the retinoid X receptor. The ligand with the highest affinity for FXR is chenodeoxycholic acid (CDCA): however deoxycholic (DCA) and cholic acid (CA) also increase BSEP expression. The co-activators ‘activating signal cointegrator-2-containing complex’ (ASCOM), ‘co-activator-associated arginine methyltransferase 1’ (CARM1) and ‘steroid receptor co-activator SRC2’ (SRC2) increase BSEP expression via FXR. Liver receptor homolog 1 (LHR-1) and nuclear factor erythroid 2-related factor (Nrf-2), a sensor for oxidative stress, also increase BSEP expression.

Expression of BSEP is regulated by a major ligand-activated transcription factor, farnesoid X receptor (FXR, NR1H4), which forms a signaling-competent nuclear receptor heterodimer with the retinoid X receptor (RXR) (Figure 2). Bile acids, such as chenodeoxycholic acid (CDCA), deoxycholic acid (DCA) and cholic acid (CA), are endogenous ligands of FXR with varying potential for activation [16,17,18,19]. Upon ligand binding, the FXR/RXR heterodimer binds to an FXR response element (FXRE) in the promoter region of BSEP, thereby inducing the expression of the transporter [20]. Additionally, components of the activating signal cointegrator-2-containing complex (ASCOM) interact with FXR to enhance BSEP expression. Ananthanarayanan and co-workers [21] showed that the recruitment of ASCOM to the BSEP promoter was disrupted in cholestasis, which was induced by common bile duct ligation. Furthermore, co-activator-associated arginine methyltransferase 1 (CARM1) also regulates FXR/RXR-dependent BSEP transcription [22]. Similarly, steroid receptor co-activator 2 (SRC2) knockout mice showed reduced expression of BSEP [23], indicating its involvement in transcriptional regulation of the transporter.

Hepatocyte-specific liver receptor homolog-1 (LRH-1, NR5A2) is another transcription factor involved in modulation of BSEP expression. LRH-1 plays a supporting role for FXR [24]. The absence of LRH-1 is associated with reduced BSEP expression and an altered BA composition, with disappearance of CA and taurocholic acid (TCA) [25]. BSEP promoter activity is also stimulated by nuclear factor erythroid 2-related factor 2 (Nrf2), a positive transcriptional regulator, which acts as a sensor for oxidative stress. Nrf2 regulates the expression of BSEP, but also that of a number of hepatic phase I and II enzymes and other hepatic efflux transporters such as MRP3 (ABCC3) and MRP4 (ABCC4) [26].

## 4. Processing and Trafficking of BSEP

Membrane insertion and folding occur at the level of the endoplasmic reticulum (ER) [27]. Insertion into the ER membrane is facilitated by the protein transport protein SEC61, which assists transmembrane portions of nascent proteins to adopt helicity prior to domain folding. Correct positioning of domains or subdomains relative to each other typically occurs late in the folding trajectory of a multidomain membrane protein. Of all ABC proteins, the folding trajectory of cystic fibrosis transmembrane conductance regulator (CFTR, ABCC7) has been studied the most [28,29]. The intracellular loops (ICLs) play a critical role in transporter folding by contributing to the formation of the functionally important TMD/NBD coupling interface [30]. Furthermore, the involvement of molecular chaperons is required, as they sense the presence of hydrophobic helices in the cytosol, and thus contribute to obtaining the folding endpoint [31].

In the ER, newly synthesized and correctly folded BSEP undergoes N-linked core glycosylation. The sugar moieties are added at four conserved asparagine residues in extracellular loop 1 (ECL1), namely Asn109, 116, 122 and 125, and then are subject to subsequent modifications while traveling through the Golgi stacks. N-linked core glycosylation in the ER lumen plays a pivotal role in ER protein folding by mediating interactions with the lectin chaperones calnexin and calreticulin and by increasing the folding efficiency [32]. Only correctly folded proteins are trafficked to the Golgi apparatus in clathrin-coated COPII vesicles. Aberrantly folded proteins are identified by the endoplasmic-reticulum-associated degradation (ERAD) machinery and retro-translocated to the cytoplasm for degradation in the 26S proteasome following ubiquitination [33]. A number of BSEP mutants, including G238V, D482G, G982R, R1153C and R1286Q, are predominantly degraded by ERAD, thus leading to a PFIC2 phenotype [27,34,35]. Different ERAD E3 ubiquitin ligases are thought to recognize and ubiquitinate different mutants of BSEP [27]. Before trafficking to the canalicular membrane, BSEP is fully glycosylated in the Golgi apparatus through trimming to the core structure and extension from the core [34]. Glycosylation directly impacts protein stability and at least two of the four glycans are required for BSEP trafficking to the canalicular membrane [35]. Using enhanced green fluorescent protein (EGFP)-tagged mouse BSEP, it was shown that the partial glycosylation of the PFIC2-related mutant D482G causes an unstable BSEP protein and reduces levels of the mature protein at the canalicular membrane [36].

The majority of integral plasma membrane proteins of polarized hepatic cells are distributed from the basolateral membrane to the appropriate apical cell surface location via transcytosis. In contrast, ABC transporters targeted to the canalicular membrane use the non-transcytotic direct route from the Golgi apparatus via Rab11a-positive apical endosomes [37,38]. Under physiological conditions, the apical pool of BSEP is strictly regulated by the demand for biliary excretion of bile salts. The intracellular endosomal pool is thought to exceed that at the canalicular membrane [39] by at least 6-fold. Internalization of BSEP is mediated by clathrin-coated vesicles and is dependent on the highly conserved endocytic cargo motif (Trp-Lys-Leu-Val) [40]. This trafficking motif is recognized by adaptor protein 2 (AP-2), which modulates the internalization process and expression of cell-surface-resident BSEP through direct interaction [41]. Moreover, trafficking of BSEP through the endosomal system to the canalicular membrane is a microtubule-dependent process and requires the myosin light chain [42], myosin Vb [43] and Rab11a. The latter two components were shown to also be associated with canalicular biogenesis by maintaining proper trafficking of Rab11a–myosin Vb-containing membranes to the canalicular membrane in polarized WIF-B9 cells [43].

Continuous cycling of BSEP between the apical and intracellular pools is disrupted in most human cholestatic liver diseases. Shifting the balance towards endocytic internalization results in impaired bile salt secretion [44]. A causative role of enhanced retrieval into the subapical endosomal compartment was demonstrated for estradiol 17 β-D-glucuronide (E17G)-induced cholestasis, an experimental model for pregnancy-related cholestasis [45,46]. In this model, BSEP was found to co-localize with clathrin, AP-2 and Rab5 as evidence for clathrin-mediated endocytosis [46]. Classical (Ca^2+^-dependent) protein kinase C (cPKC)–p38, mitogen-activated protein kinase (MAPK) and phosphoinositide 3-kinase (PI3K)–ERK1/2 signaling pathways are thought to be involved in (E17G)-induced cholestasis [47,48,49].

TCA, the major bile acid in mammals, as well as cyclic adenosine monophosphate (cAMP) are known to increase the apical pool of BSEP within minutes by promoting its cellular relocation [37]. Moreover, TCA was demonstrated to induce the formation of bile canaliculi in mice via the liver kinase B1 (LKB1)–AMP-activated protein kinase (AMPK) pathway [50]. A subsequent publication showed that knocking out LKB1, the upstream serine–threonine kinase, which is implicated in regulation of cellular energy metabolism, impairs both canalicular biogenesis and intracellular trafficking of BSEP. On the other hand, cAMP induces BSEP trafficking through a PKA-mediated pathway, which does not involve AMPK activation [51]. Unlike TCA-mediated trafficking, this process is PI3K-independent [52]. Similar to TCA, the conjugated bile salt tauroursodeoxycholate (TUDCA) also promotes the relocation of BSEP to the canalicular membrane through activation of the p38 MAPK [53,54]. Similar to other conjugated bile salts, TUDCA stimulates the ATPase activity of BSEP [55].

Ubiquitination is another modification, which changes the expression of cell-surface-resident BSEP. The half-life of BSEP in the canaliculi is shortened by modification with two to three ubiquitin molecules. This induces the removal of the protein from the cell surface, whereby the rates are governed by the degree of ubiquitination. While the PFIC2-related mutations E297G and D482G cause short-chain ubiquitination, thereby shortening the half-life of cell-surface-resident BSEP, the chemical chaperone 4-phenylbutyrate (4-PB) reduces its degradation rate [56]. In a later study, ubiquitination of canalicular BSEP was shown to act as a signal for internalization by promoting clathrin-mediated endocytosis. After internalization, BSEP is either recycled back to the canalicular membrane in a Rab11-dependent manner or degraded through a ubiquitination-independent pathway [57]. Degradation was suggested to be lysosome-mediated and dependent on a sorting signal from within the endosomal compartment [58].

## 5. Structural Models of BSEP

Before publication of the cryo-electron microscopy (cryo-EM) structure of BSEP [59], several homology models of the transporter were presented. Kubitz and colleagues [60,61] generated an outward-facing homology model of ABCB11 by using the Sav1866 structure (PDB: 2HYD [62]) as a template. This model was used to show a putative antibody binding site at the long ECL1, as well as to indicate positions of disease-causing mutations. The model compares well at the individual domain level with the cryo-EM structure that has recently become available. Giovannoni et al. [63] created a model based on the corrected mouse ABCB1 structure (PDBID: 4M1M [64]). Here, loops that could not be matched to the template were not modeled (ABCB11 residues 102–120 and 659–728). The model was used to localize positions of disease-causing mutations. Again, this model agrees well with the cryo-EM structure within the resolution at which cryo-EM data were presented. Notably, an overall structural alignment of mouse ABCB1 (PDBID: 4M1M [64]) and the cryo-EM structure of ABCB11 results in an RMSD of 0.36 nm, with a better fit of the TMDs. Dröge et al. [65] also used the mouse ABCB1 structure as a template. This model was used to locate the positions of the most commonly occurring PFIC2 missense mutations. Moreover, in the process of structure evaluation, different web services were used to predict the influence of missense mutations on protein function. The authors provided a list of possible effects of newly identified mutations included in their study. However, the accuracy of this prediction was not evaluated. In a different study, Jain and co-workers [66] generated an ABCB11 homology model also using the inward-facing mouse ABCB1 structure as the template (PDB: 4M1M [64]). Extensive docking studies with 405 inhibitor compounds and 807 non-inhibitors were performed in order to explore the interaction with small molecules. Prediction accuracy results of 81% in the training set and 73% in two external test sets were obtained. In addition to standard scoring functions, the homology model used for docking was validated by molecular dynamics (MD) simulation. The use of MD simulations in protein stability checks is a well-established procedure, however it is computationally expensive. Short simulations were performed for structure validation of membrane-inserted ABCB11 homology models. We also previously [67] generated a homology model based on the X-ray structure of Sav1866 as a template (PDB ID: 2ONJ [62]) to elucidate the NBD–NBD interdomain communication of the transporter. The model allowed us to infer the potential roles of conserved motifs of the nucleotide-binding domains in ATP hydrolysis and the transmission of conformational changes from the NBDs to the TMDs.

In 2020, Wang et al. [59] determined the human BSEP structure using cryo-EM (PDBID: 6LR0). This structure has an average resolution of 0.35 nm, with the TMDs being resolved at 0.33 nm. The protein shows an inward open state in the absence of nucleotides or other small molecules. According to this structure, ABCB11 closely resembles ABCB1, with the most similar structure found in the Protein Data Bank [68] being the apo inward-open ABCB1 structure (PDBID: 6GDI, RMSD: 0.206 nm [69]. Thus, it shares the typical type I exporter fold with the other members of the ABCB subfamily. The domain-swapped transmembrane helices are connected by coupling helices 2 and 4, which are embedded in grooves formed between the core and the helical domain of the NBDs, supporting their crucial role in interdomain communication. Interestingly, inside the central cavity, contiguous electron density was found, into which the N-terminus of the protein could be fitted. Currently, no biochemical data are available with respect to any putative auto-inhibition of BSEP by this N-terminus. The poor resolution of ECL1 (Q101-I134) reflects its highly dynamic nature. It contains the four known glycosylation sites (N109, 116, 122 and 125) [70].

BSEP harbors two ATP-binding sites, one of which is canonical and capable of ATP hydrolysis. When compared to ABCB1, only four amino acids differ in NBS1. These are E502, M584, R1221 and E1223 in BSEP corresponding to S474, E556, G1178 and Q1180 in the ABCB1 protein sequence [71]. The amino acid changes result in a catalytically inactive ATP binding site (NBS1), which is also variably called “degenerate” NBS. It has been suggested that the degenerate site imparts extended functionality to the transporter. The mechanistic details are currently missing, although the role of ATP hydrolysis in each of the two NBDs has been elucidated in greater detail [67].

## 6. Experimental Model Systems

In vitro and in vivo models have been developed for the study of BSEP function, folding and trafficking, as well as the actions of drug candidates with the potential to treat the malfunction or incorrect cellular routing of the transporter. These model systems are discussed below with respect to their potentials and limitations.

### 6.1. In Vitro Models

#### 6.1.1. Membrane Vesicles

For the study of substrate transport and inhibition by drugs and metabolites, membrane vesicles represent the most commonly used model system. Vesicles are either prepared from BSEP-transfected insect cell lines (Sf9 and Sf21), which give higher protein yields, or mammalian cell lines (including CHO, HeLa, MDCK, LLC-PK1 and HEK cells). Despite having lower protein expression, mammalian cells are often preferred for functional studies, as insect cells show a different lipid membrane composition and only core glycosylated protein is produced. In order to overcome lower expression levels in mammalian cells, the Bac/Mam gene transfer system has been advocated [72]. For experimental details on the preparation of membrane vesicles, readers are referred to [13,69,73]. As a mixture of inside-out and right-side-out vesicles is obtained, a protocol for increasing the yield of inside-out vesicles has been published [74]. In addition, a protocol for preparation of membrane vesicles from canalicular membranes of rat hepatocytes has been reported [73]. These vesicles were used for the identification and characterization of BSEP substrates and inhibitors [75,76].

High-quality membrane vesicles represent an ideal experimental system for transport and transport inhibition studies, but cannot be used to address aspects of trafficking or cellular metabolism [77]. As in inside-out vesicles, the BSEP NBDs are exposed towards the medium, ATP and substrates can be added into the transport medium and accumulation of substrates in vesicles can be monitored by rapid filtration. We previously used membrane vesicles from plasmid-transfected HEK cells to study the domain interaction and the roles of the canonical and non-canonical NBS in supporting BSEP substrate transport [67].

#### 6.1.2. Polarized Cell Lines Expressing BSEP

Polarized MDCK and LLC-PK1 cells have been used to study BSEP function in intact cells. The experimental system requires double transfection with BSEP and the hepatocyte bile salt uptake transporter NTCP, as BSEP-mediated efflux can only be monitored after bile salt substrates have been taken-up into cells. Physiologically, NTCP enables the reentry of bile salts from the circulation into hepatocytes in the context of the enterohepatic circulation of these compounds between the intestine and the liver. Polarized cells are grown on a permeable membrane in a hang-in assembly. BSEP is localized on the apical surface. The function of BSEP is determined from the ratio of basal-to-apical as compared to apical-to-basal transport of substrates [78,79].

#### 6.1.3. Primary Hepatocyte Cultures

When cultured in the appropriate medium, hepatocytes form tight junctions to generate sealed tube-like structures, which resemble bile canaliculi [80,81]. Therefore, this system provides the possibility of assessing the excretion of drugs and bile components into bile canaliculi [82,83,84]. Hepatocytes in suspension also provide a suitable option for studying drug metabolism and drug transport [85,86]. One major advantage of hepatocyte suspension cultures is their easy, quick and high-yield preparation. Furthermore, this assay does not require radiolabeled substrates [87]. Therefore, this system is often used for large-scale screening of drugs for assessment of drug-induced liver injury (DILI). It has to be kept in mind, however, that the presence of multiple transporters in primary hepatocytes limits their use for assessing BSEP-specific substrates [88].

### 6.2. BSEP Knockout Animals

#### 6.2.1. Rodents

In order to investigate the mechanisms involved in innate and acquired intrahepatic cholestasis, BSEP knockout animal models (mice and rat) have been established [89,90]. Recently, the CRISPR/cas9 technology has been employed to knock out the BSEP gene in adult mice [91,92]. In all models, expression of BSEP was strongly reduced, thus providing an alternative experimental model for studying intrahepatic cholestasis and putative therapeutic intervention in rodents. Interestingly, the mouse models do not show signs of severe cholestasis as seen in humans, because these mice produce a large amount of poly-hydroxylated bile acids, which are excreted renally [93]. Wang et al. used this model system to suggest that P-glycoprotein (ABCB1) can act as a compensatory bile salt transporter, which alleviates the severity of cholestasis in BSEP knockout mice [94].

#### 6.2.2. Zebrafish

Recently, Ellis et al. generated an abcb11b knockout zebrafish by using the CRISPR/Cas-9 gene editing technology [95]. Abcb11b is the orthologue of the human BSEP gene in zebrafish. The histological and ultrastructural analysis showed a morphological hepatocyte injury pattern similar to that seen in patients with PFIC2. Similar to the situation in humans, BSEP deficiency induced autophagy in zebrafish hepatocytes. Treatment with rapamycin restored bile acid excretion, attenuated hepatocyte damage and extended the life span of abcb11b mutant zebrafish. These effects were paralleled by a recovery of the correct canalicular localization of multidrug resistance protein 1.

Due to the transparency of these fish, the system allows monitoring of the bile flow in the intact animal with fluorescently labeled bile salt analogs. Furthermore, P-glycoprotein, which is reported to play a compensatory role (by transporting bile acids, and thereby protecting the hepatocytes from cholestasis induced injury) in BSEP knockout mice [96], is mislocalized to the hepatocyte cytoplasm in mutant zebrafish.

Animal models play an important role in studying cholestatic liver disease [93]. In contrast, identification of drug candidates for the treatment of folding and functionally deficient BSEP-mutants usually relies on in vitro model systems. For such studies, the patient-specific mutations are generated and expressed in cell lines. The impact of small molecules on the structure and function of BSEP is evaluated. Once the drugs have proven a potential in cell models, they are translated to a clinical setting [97,98].

Hydrodynamic tail vein injection in combination with the CRISPR/Cas9 technology has been used to specifically delete BSEP in mice and to study the consequences of the loss of an enzyme of the urea cycle (argininosuccinate lyase) [91]. In a similar way, such a BSEP knockout model system may be used for the study of mice expressing mutant forms of human BSEP in the liver and to monitor the effects of drug candidates on the folding, trafficking and function of the transporter.

## 7. Treatment Options for BSEP-Related Diseases

**Figure 3:**
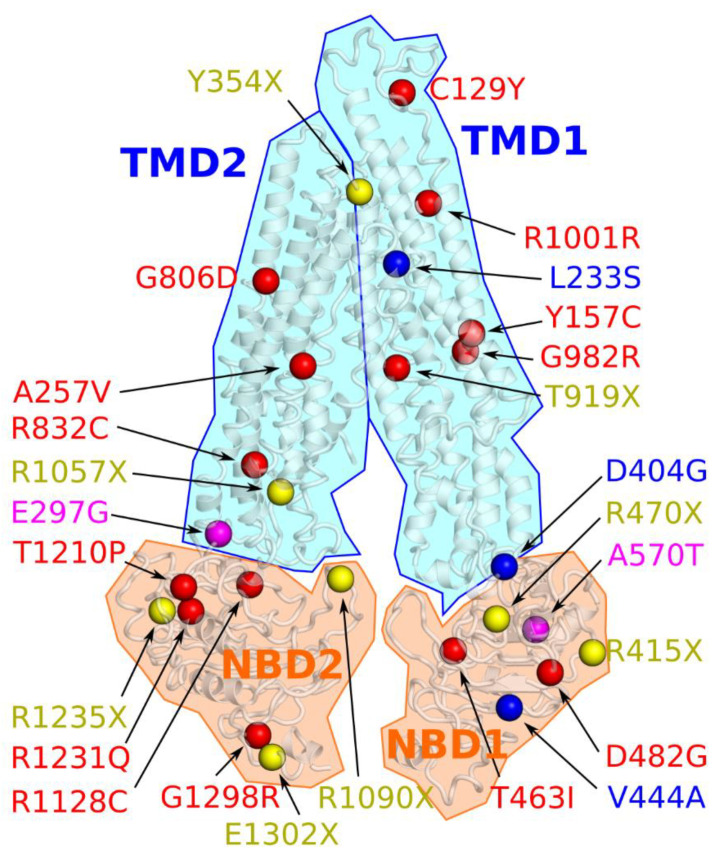
The positions of disease-causing mutations contained in Table 1 are shown in the ABCB11 cryo-EM structure. C-alpha atoms are shown as spheres and colored according to the resulting disease phenotype (PFIC2: red; BRIC2: blue; either PFIC2 or BRIC2: magenta; nonsense mutations: yellow). Outlines and filled colors indicate the domain organization of the transporter in accordance with Figure 1.

Impairment in the expression or function of BSEP leads to one of three human disease phenotypes of differing severity: PFIC2, BRIC2 and intrahepatic cholestasis of pregnancy (ICP). Several drugs with the potential to enhance the expression and function of the transporter have been reported. Disease-causing mutations and potential correctors are listed in Table 1 and depicted in the ABCB11 cryo-EM structure in Figure 3.

### 7.1. Transcriptional Modulators

FXR is the major ligand-activated transcription factor controlling BSEP expression, which makes it a possible target for therapeutic intervention. Furthermore, 6α-ethyl-CDCA (obeticholic acid, OCA), a derivative of the primary human bile acid CDCA and an FXR agonist, was approved by the FDA for the treatment of primary biliary cholangitis (PBC) either in monotherapy or in combination with UDCA in adults, depending on UDCA responsivity and tolerability [120]. OCA’s 100-fold higher FXR-activating potential (as compared to the natural ligand CDCA) formed the basis for advocating it as a novel therapeutic treatment strategy for PBC [121]. Its long-term efficacy and safety profile were reported recently [122]. OCA was also introduced for the treatment of non-alcoholic steatohepatitis (NASH), whereby the interim analysis from a phase 3 trial demonstrated clinical improvement and partial reversal of histopathological features [123]. Several other steroidal and non-steroidal FXR agonists, such as EDP-305 and tropifexor (LJN4524), are currently being investigated in a clinical setting for the treatment of NASH [124].

Garzel and co-workers evaluated the effects of 30 BSEP inhibitors on BSEP expression and FXR activation in human primary hepatocytes to understand the underlying mechanisms of drug-induced liver injury (DILI) [125]. Among five potent transcriptional repressors, lopinavir and troglitazone were shown to mediate their effects by reducing the FXR activity. The latter drug was previously withdrawn from the market because of DILI [125]. A number of natural FXR agonists or antagonists were reported to modulate FXR activity in a variety of model systems, as reviewed in detail by Hiebl et al. [126]. A natural product, geniposide, was reported to modulate the expression of BSEP via the FXR, as well as via the Nrf2 signaling pathways [127]. On the other hand, 9-cis retinoic acid (9cRA) is an RXR agonist, which when co-administered with CDCA, represses FXR/RXR-mediated expression of BSEP, thus exerting an opposite effects on BSEP transcription [128].

### 7.2. Ursodeoxycholic Acid (UDCA)

UDCA is one of the most commonly used agents for the treatment of cholestatic disorders. It showed promising results in animal models and in patients by alleviating disease symptoms. Although the exact mechanism of action of UDCA is not known, it was reported that the compound may act by correcting a potential trafficking deficiency of BSEP mutants, as well as by reducing the internalization of the transporter [112,129,130]. Furthermore, UDCA also reduces the overall hydrophobicity of the bile acid pool, thereby protecting hepatocytes from damage [131]. However, in some patients UDCA failed alleviate disease symptoms. Likely this finding reflects the genetic diversity of the underlying disease [129].

A UDCA derivative, norUDCA, is also being used for treatment of BSEP-related diseases. Because of its capacity for cholehepatic shunting (i.e., bypassing the normal enterohepatic circulation), norUDCA counteracts bile duct damage via bicarbonate-rich choleresis. Furthermore, norUDCA has antifibrotic, antiproliferative and anti-inflammatory properties and propagates bile acid detoxification through elimination via the urine [131,132,133].

### 7.3. Chemical Correction with 4-PB

A large number of mutations have been reported to interfere with either BSEP folding or its correct trafficking to the canalicular membrane. Among these are the E297G and D482G mutations, which account for approximately 60% of PFIC2 cases in the European population [119]. The underlying hypothesis behind the concept of chemical correction is that these mutants, when rescued to the canalicular membrane, would function normally, and thus the disease phenotype would be alleviated. Furthermore, 4-PB, an FDA-approved drug for the treatment of urea cycle disorders, functions as a chemical chaperone for folding-deficient BSEP variants [56]. Indeed, in vitro studies in HEK293 and MDCKII cell lines indicated that upon treatment with 4-PB, these mutants would show enhanced surface expression, as well as increased TCA transport activity [103,104]. Furthermore, in support of the validity of this concept, 4-PB treatment also increased the biliary excretion of TCA in animal models [104].

Recently, use of 4-PB showed promising results in a clinical setting. Gonzales and co-workers [97,98] showed that treating PFIC2 patients carrying at least one mutation (out of p.G982R, p.R1128C and p.T1210P) with 4-PB led to an improvement in serum liver parameters, including the serum bile acid concentration, and a reduction in the pruritus score. Similarly, a preterm infant diagnosed with BSEP-related cholestasis was treated with 4-PB and showed an improvement of the disease symptoms [134]. In addition, BRIC2 patients have also been treated successfully with 4-PB [109]. The therapeutic doses ranged from 150 to 500 mg/kg/day. In some patients, a 4-PB dose of ≤ 350 mg/kg/day had no beneficial effect, while a high dose regimen (500 mg/kg/day) improved disease symptoms [109,117]. In a recent study, two PFIC II patients were given a combination of 4-PB, oxcarbazepine (a peripheral nerve stabilizer reducing pruritus) and maralixibat (an apical sodium-dependent bile acid transporter inhibitor), which had a beneficial effect on disease markers [101]. Most of these studies did not show apparent side effects, even in the high-dose regimen. However, psychological disorders (bipolar and related disorders) have been connected to the use of 4-PB in a clinical setting [135].

### 7.4. Potentiation with Ivacaftor

UDCA and 4-PB have been shown to correct the misfolding of trafficking-deficient variants. However, several disease-associated mutations (especially those in the NBDs) do not interfere with folding, trafficking and canalicular localization, but rather lead to impairment of transporter function [65,119]. Ivacaftor (VX-770) a potentiator has been approved by the FDA for treatment of some class III (gating deficient) CFTR mutants [136,137]. In these variants, an improvement of respiratory function could be demonstrated. Similar to clinical results in CFTR patients, ivacaftor was shown to rescue the function of missense mutations in the NBDs of ABCB4/MDR3 [138]. Recently, Mareux and co-workers showed that ivacaftor also rescued the function of an NBD missense mutation (T463I) of BSEP [110]. The mechanism of action of ivacaftor presently remains unclear.

### 7.5. Readthrough Therapy with Gentamicin

Nonsense mutations result in an in-frame premature termination codon and the absence of functional protein. This results in severe phenotypes of the disease and an increased risk for the development of hepatocellular carcinoma [139]. The aminoglycoside antibiotic gentamicin binds to ribosomes and induces a translational readthrough at the premature termination codon, thereby leading to fractional restoration of synthesis of the full-length protein [140,141]. In a recent study, Amzal et al. [118] evaluated the impact of gentamicin on six BSEP nonsense mutations (Y354X, R415X, R470X, R1057X, R1090X and E1302X) in vitro. Readthrough results were significantly increased for all mutations. The strongest responses were seen for the R1090X mutation, with partial restoration and correct localization at the plasma membrane of HepG2 and Can 10 cells. The rescued protein was shown to mediate transcellular transport of [^3^H]TC in MDCK cells. Expression of the R1090X mutant was shown to be further enhanced by simultaneous treatment with 4-PB [118].

## 8. Summary and Conclusions

Despite the relative infrequency of the inherited forms of progressive intrahepatic cholestasis, the symptoms are severe, and about half of the patients progress to a stage of the disease that makes them candidates for liver transplantation. Therefore, the quest for identification of causal therapies that go beyond the purely symptomatic treatment of pruritus is an important objective. Attempts to alleviate disease symptoms by transcriptional upregulation are directed towards missense mutations with preserved functionality. Similarly, those mutants that are folding- and consequently trafficking-deficient have successfully been treated with folding correctors. Recent evidence points to yet another therapeutic path, in which the function of impaired BSEP mutants is potentiated by drugs that were initially developed to treat different disease entities, which are also associated with a malfunction of human ABC proteins. However, BSEP missense or deletion mutations and mutants with compromised functionality will only be amenable to therapy using gene editing. Current advances in gene editing technologies have not been considered in this review, as they are subject to another article in this Special Issue. The availability of the recently published cryo-EM structure of BSEP can be considered an important basis for structure-based drug design. Moreover, structural data, MD-simulations and site-directed mutagenesis studies continuously expand our understanding of the functional biology of BSEP.

We also briefly summarized available in vitro model systems for the functional characterization of BSEP, animal models and case reports discussing emerging clinical therapies. The perspective that treatment regimens combining small molecules with different mechanisms of action will ultimately lead to an improvement in the quality of life and life span of affected individuals appears promising.

## Figures and Tables

**Table 1:** Synopsis of a subset of disease-causing mutations, for which potential therapeutic interventions have been proposed. The defects, model systems and associated disease phenotypes are indicated. With the exception of the C129Y and G806D variants, the listed mutants represent a subset of a total of 192 disease-associated missense or nonsense mutations that have been identified to date [99]. PFIC2: progressive familial intrahepatic cholestasis type 2, BRIC2: benign recurrent intrahepatic cholestasis type 2,4-PB: 4-phenylbutyrate, CA: cholic acid, CDCA: chenodeoxycholic acid, DCA: deoxycholic acid, UDCA: ursodeoxycholic acid, FXR: farnesoid X receptor.

Nucleotide Change	Type of Mutation	Amino Acid	Defect	Potential Corrective Therapy	Cell Line/Organism	Disease	References
c.386GA	Missense	C129Y ^1^	Impaired membrane trafficking, reduced level of mature protein	4-PB	HEK293T	PFIC2	[100]
c.470AG c.3892GA	Missense	Y157C G1298R	Reduced/absent BSEP activity	4-PB in combination with oxcarbazepine and maralixibat	Patient with 2 heterozygous missense mutations	PFIC2	[101]
c.698TC	Missense	L233S	-	Methylprednisolone	Patient with heterozygosity in ABCB11, as well as in CFTR, NPHP4 and A1ATD	BRIC2	[102]
c.890AG	Missense	E297G ^2^	Protein instability, ubiquitin-dependent degradation [103], impaired membrane trafficking, reduced level of mature protein	4-PB	Madin-Darby canine kidney (MDCK) II cells and Sprague–Dawley rats	BRIC2, PFIC2	[104]
				Glycerol, glycerol at 28 °C	CHO-K1 cells		[105]
				CA, CDCA, DCA, UDCA, GW4064 (FXR agonist)	MDCK II cells		[106,107]
				Butyrate and octanoic acid	MDCK II cells		[108]
c.1211AG	Missense	D404G	Reduced level of mature protein, ER-like distribution	4-PB	HEK293T cells	BRIC2	[109]
c.1211AG c.1331TC	Missense	D404G V444A	Reduced level of mature protein	4-PB	Patient compound heterozygous for D404G and homozygous for V444A mutations	BRIC2	[109]
c.1388CT	Missense	T463I	Impaired ATP-binding, BSEP dysfunction	Ivacaftor	MDCK II cells	PFIC2	[110]
c.1445AG	Missense	D482G ^2^	Protein instability, ubiquitin-dependent [103], impaired membrane trafficking, reduced level of mature protein, severe differential splicing [105]	4-PB	MDCK II cells and Sprague– Dawley rats	PFIC2	[104]
				Sodium butyrate and 4-PB	HEK293T cells		[103]
				Butyrate and octanoic acid	MDCK II cells		[108]
c.1708GA	Missense	A570T	Reduced level of mature protein, reduced BSEP activity [105]	UDCA	MDCK II cells	BRIC2 PFIC2	[111]
				Glycerol at 28 °C	CHO-K1 cells		[105]
c.2417GA	Missense	G806D	Reduced level of mature protein, aberrant splicing	4-PB	BSEP-deficient hepatocyte-like cells	PFIC2	[112]
c.-24CA	5′-UTR (five prime untranslated region)						
c.2494CT	Missense	R832C	Differential splice products [105]	Steroid	Patient with compound heterozygosity	PFIC2	[113]
c.150+3AC	Splice-site mutation		Partial exon skipping [114]				
c.2756_2758delCCA	Deletion	T919del	Reduced BSEP activity [115]	Steroid	Patient with compound heterozygosity	PFIC2	[113]
c.3703CT	Nonsense	R1235X	Truncated, non-functional transporter [115]				
c.2944GA	Missense	G982R	Retention in ER, reduced level of mature protein	UDCA, 4-PB single agents or in combination	Can 10 cells	PFIC2	[97]
c.2944GA	Missense	G982R	Retention in ER, reduced level of mature protein	4-PB	Patient with compound heterozygosity	PFIC2	[97]
c.770CT	Missense	A257V	Normal canalicular expression of BSEP				
c.2944GA	Missense	G982R	Retention in ER, reduced level of mature protein	4-PB	Patient with compound heterozygosity	PFIC2	[97]
c.3003AG	Silent	R1001R	Abnormal splicing [116]				
c.3382CT	Missense	R1128C	Retention in ER, reduced level of mature protein, Mild exon skipping [105]	UDCA, 4-PB single agents or in combination	Can 10 cells	PFIC2	[97]
				4-PB	Patient homozygous for R1128C		
c.3628AC	Missense	T1210P	Retention in ER, reduced level of mature protein	UDCA, 4-PB single agents or in combination	Can 10 cells	PFIC2	[97]
				4-PB	Can 10 cells		[98]
				4-PB	Patient with homozygous mutation		[97,98]
c.3692GA	Missense	R1231Q	Retention in ER [117], no splicing, immature protein [105]	4-PB	HEK293T cells, McA-RH7777 cells, patient with homozygous mutation	PFIC2	[117]
c.1062TA	Nonsense	Y354X	Premature termination codon	G418, gentamicin	NIH3T3 cells (increased readthrough)	PFIC2	[118]
c.1243CT	Nonsense	R415X	Premature termination codon	G418, gentamicin	NIH3T3 cells (increased readthrough)	PFIC2	[118]
				Gentamicin	HEK293 cells (production of a full-length BSEP protein)		
c.1408CT	Nonsense	R470X	Premature termination codon	G418, gentamicin	NIH3T3 cells (increased readthrough)	PFIC2	[118]
				Gentamicin	HEK293 cells (production of a full-length BSEP protein)		
c.3169CT	Nonsense	R1057X	Premature termination codon	G418, gentamicin	NIH3T3 cells (increased readthrough)	PFIC2	[118]
				Gentamicin	HEK293 cells (production of a full-length BSEP protein)		
c.3268CT	Nonsense	R1090X	Premature termination codon	G418, gentamicin	NIH3T3 cells (increased readthrough)	PFIC2	[118]
				Gentamicin	HEK293, Can10 and HepG2 cells (production of a full-length BSEP protein and localization at the PM of HEK293 and at the CM of Can 10 and HepG2 cells)		
				Gentamicin treatment with UDCA, 4-PB and UDCA + 4-PB, gentamicin at 27 °C	Can10 cells (increased canalicular expression)		
				Gentamicin, gentamicin with 4-PB, gentamicin at 27 °C	NTCP expressing MDCK cells (significantly increased transport of [3H]TC)		
c.3904GT	Nonsense	E1302X	Premature termination codon	G418, gentamicin, PTC124	NIH3T3 cells (increased readthrough)	PFIC2	[118]

Note: ^1^ Most frequently reported in Japan. ^2^ E297G and D482G mutations account for 58% of PFIC2 cases in the Western population [119].

## Data Availability

Not available.

## Abbreviations

ABC	ATP-binding cassette
AMPK	AMP-activated protein kinase
AP-2	adaptor protein 2
ASCOM	activating signal cointegrator-2-containing complex
BSEP (ABCB11)	bile salt export pump
BRIC2	benign recurrent intrahepatic cholestasis type 2
CA	cholic acid
cAMP	cyclic adenosine monophosphate
CARM1	co-activator-associated arginine methyltransferase 1
CFTR (ABCC7)	cystic fibrosis transmembrane conductance regulator
CDCA	chenodeoxycholic acid
CM	canalicular membrane
cPKC	classical (Ca2+-dependent) protein kinase C
cryo-EM	cryo electron microscopy
DCA	deoxycholic acid
DILI	drug-induced liver injury
ECL	extracellular loop
Epac	exchange protein directly activated by cAMP
ICL	intracellular loop
ICP	intrahepatic cholestasis of pregnancy
ERAD	Endoplasmic-reticulum-associated degradation
E17G	estradiol 17 β-D-glucuronide
FXR (NR1H4)	farnesoid X receptor
FXRE	FXR response element
LKB1	liver kinase B1
LRH-1 (NR5A2)	liver receptor homolog-1
MAPK	Mitogen-activated protein kinase
MRP3 (ABCC3)	Multidrug-resistance-associated protein 3
MRP4 (ABCC4)	Multidrug-resistance-associated protein 4
NASH	non-alcoholic steatohepatitis
NBD	nucleotide-binding domain
NBS	nucleotide-binding site
Nrf2	nuclear factor erythroid 2-related factor 2
NTCP (SLC10A1)	sodium taurocholate co-transporting polypeptide
OCA	6α-ethyl-CDCA (obeticholic acid)
4-PB	4-phenylbutyrate
PBC	primary biliary cholangitis
PFIC2	progressive familial intrahepatic cholestasis type 2
PI3K	phosphoinositide 3-kinase
PM	plasma membrane
RXR	retinoid X receptor
SRC2	steroid receptor co-activator 2
TCA	taurocholic acid
TMD	transmembrane domain
TUDCA	tauroursodeoxycholic acid
UDCA	ursodeoxycholic acid
